# Primary hyperoxaluria: insights into its clinical presentation, genetic mutations, and transplantation outcomes in a pediatric population in a tertiary care center

**DOI:** 10.1186/s13023-025-04082-8

**Published:** 2025-10-28

**Authors:** Bayan Sayed, Raghad Alhuthil, Sermin Saadeh, Turki Al-Shareef, Ibrahim Alhassoun, Essam Al-Sabban

**Affiliations:** Department of Pediatrics, King Faisal Specialist Hospital and Research Centre, Riyadh, 11211 Saudi Arabia

**Keywords:** *AGXT* gene, Nephrocalcinosis, Liver transplantation, Chronic kidney disease

## Abstract

**Background:**

Primary hyperoxaluria (PH) is a rare inherited disorder characterized by excessive oxalate accumulation in blood and urine due to defects in glyoxylate metabolism, leading to significant clinical consequences. As genetic and phenotypic heterogeneity contribute to the morbidity of PH, this study examined the phenotypes and genotypes of PH among confirmed pediatric patients (< 18 years) diagnosed with PH at a tertiary care center in Saudi Arabia between 2014 and 2023.

**Results:**

Twenty-one patients from 14 families, including 10 boys (47.6%) and 11 girls (52.4%), were included. Six families had more than one affected child. The median age at diagnosis was 36 months (interquartile range = 6–84). All but one patient had PH type 1 [due to alanine–glyoxylate aminotransferase gene variant (*AGXT*)], with the remaining patient having PH type 2 [associated with glyoxylate and hydroxypyruvate reductase gene (GRHPR)]. The most frequent variant in the *AGXT* gene was NM_000030.3: c.33dup (p.Lys12GlnfsTer156), which was detected in 38.1% of patients. High parental consanguinity (90.5%) and positive family history (81%) were notable. The major clinical manifestations were kidney stones (71.4%), nephrocalcinosis (47.6%), failure to thrive (38.1%), stage V chronic kidney disease (42.9%), and hematuria (33.3%). All patients received conservative management, in addition to liver transplantation in eight patients and combined liver–kidney transplantation in two patients. The mortality rate was 14.3% (3/21). *AGXT* variants such as NM_000030.3:c.481G > A(p.(Gly161Ser) and NM_000030.3: c.346G > A (p.(Gly116Arg) displayed potential trends with worse renal prognosis.

**Conclusions:**

PH type 1 is the predominant form of hyperoxaluria among Saudi patients. High consanguinity might contribute to its disease burden. Early diagnosis and intervention are critical for improving outcomes. Recently approved RNA interference-based therapies offer promising outcomes, potentially reducing the need for organ transplantation in patients with PH type 1.

**Clinical trail number:**

Not applicable.

**Supplementary Information:**

The online version contains supplementary material available at 10.1186/s13023-025-04082-8.

## Background

Primary hyperoxaluria (PH) is a rare inherited disorder characterized by oxalate overproduction in blood and urine due to defects in glyoxylate metabolism (see supplementary file 1), leading to recurrent kidney stones, nephrocalcinosis, and progressive kidney damage. Among its three known subtypes, namely PH type 1 (PH1), PH2, and PH3. PH1 is the most severe and commonly encountered, particularly in children, in whom it can rapidly progress to end-stage renal disease if left untreated [1, 2]. The prevalence of PH is not well documented in Saudi Arabia; however, a prevalence of 1:149,000 has been reported in Europe [2].

PH1 is caused by mutations in the alanine–glyoxylate aminotransferase gene (*AGXT*), which encodes the liver-specific enzyme alanine–glyoxylate aminotransferase (AGT). AGT deficiency or dysfunction leads to the accumulation of glyoxylate and its conversion into oxalate, contributing to calcium oxalate crystal formation and kidney injury [3, 4]. More than 150 pathogenic variants of *AGXT* have been identified, including population-specific variants such as c.33dupC and c.815_816insGA [5, 6]. Responsiveness to pyridoxine (vitamin B6) has been linked to these variants [7, 8].

The relationship between the genotype and phenotype in PH1 has important clinical implications. Certain variants are associated with milder forms of the disease or a delayed progression to renal failure, whereas others can predict early-onset, severe outcomes such as infantile oxalosis, a life-threatening condition marked by systemic oxalate deposition [9, 10]. Factors such as nephrocalcinosis, recurrent nephrolithiasis, and early age at presentation have also been linked to a higher risk of kidney failure, particularly in children [4, 5].

Although PH remains rare globally, understanding regional variations in its genetic and clinical presentation is essential, especially in populations with high rates of consanguinity, such as Saudi Arabia, where recessive genetic disorders might be more common and distinct mutation patterns can emerge [11]. However, local data on PH in Saudi Arabia are limited [12, 13].

This case series investigated the genotypes and clinical phenotypes in Saudi patients with confirmed PH. By highlighting genetic variations and disease presentations, the study aimed to contribute to both national and international efforts to improve the early diagnosis, risk stratification, and management of this challenging disorder.

## Methods

This retrospective case series was conducted at the King Faisal Specialist Hospital and Research Centre (KFSHRC) in Riyadh, Saudi Arabia, focusing on pediatric patients diagnosed with PH between 2014 and 2023.

Individuals younger than 18 years at the time of diagnosis were eligible. Patients with incomplete phenotypic or genotypic data were excluded from the study. The diagnosis of PH was based on the combination of clinical presentation, biochemical markers, and genetic confirmation [14, 15]. Conservative treatments, including hyperhydration (2–3 L/m^2^ body surface area in children daily), alkalization (0.1–0.15 g/kg Potassium citrate in patients with preserved kidney function), and pyridoxine, were employed in most patients.

Data were collected from patients’ medical records and entered into the REDCap database. The extracted variables included demographic information, medical history, laboratory findings, and genetic test results.

After obtaining consent from the parents and/or caregivers of the patients, genetic testing was performed using either targeted panels or whole-exome sequencing. DNA was extracted from peripheral blood samples. The clinical significance of the identified variants was interpreted according to the American College of Medical Genetics and Genomics guidelines [16].

Descriptive statistics were conducted using STATA version 18 (StataCorp, College Station, TX, USA). Ethical approval was granted by the ethics committee at KFSHRC (Reference Number: 2231334).

## Results

**Fig. 1:**
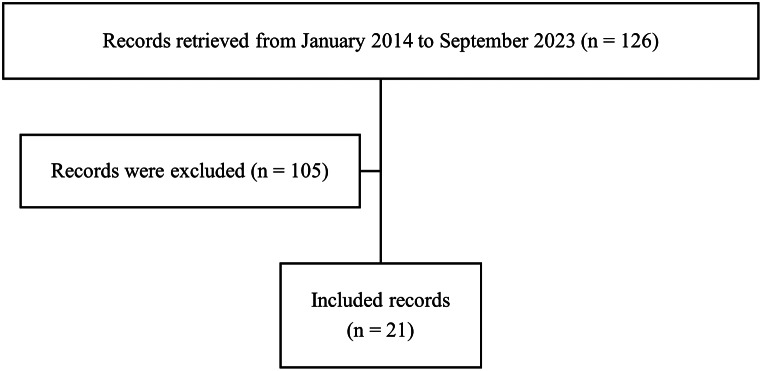
Flowchart of subjects’ enrollment

Of the 126 records reviewed, 20 patients from 14 families with a diagnosis of PH1, and one case with PH2 were included in the analysis (Fig. 1). Six families had more than one affected child (see Supplementary File 2). The cohort included 10 boys and 11 girls. The median age at enrollment was 10 years [interquartile range (IQR) = 6–13], whereas the median age at diagnosis was 36 months (IQR = 6–84). PH1 was the most prevalent form, identified in 95.2% of patients. High rates of consanguinity (90.5%) and positive family history of PH (81%) were observed (Table 1).

**Table 1:** Background information of 21 patients with PH from 14 families

Characteristics	*n* (%) or median [IQR]
**Gender**	
Male	10 (47.6)
Female	11 (52.4)
**Current age (years)**	10 [6–13]
**Age at diagnosis (months)**	36 [6–84]
**Consanguinity**	19 (90.5)
**Positive family history**	17 (81.0)
**PH type**	
Type 1 (*AGXT* mutations)	20 (95.2)
Type 2 (*GRHPR* mutations)	1 (4.8)
**Clinical features**	
Kidney stones	15 (71.4)
UTI	3 (14.3)
Hydronephrosis	2 (9.5)
Stage I CKD	0 (0.0)
Stage II CKD	2 (9.5)
Stage III CKD	4 (19.0)
Stage IV CKD	0 (0.0)
Stage V CKD	9 (42.9)
Nephrocalcinosis	10 (47.6)
Failure to thrive	8 (38.1)
Hematuria	7 (33.3)
Proteinuria	0 (0.0)
Dysuria	1 (4.8)
**Biochemical findings**	
Serum oxalate level (ref.: 11–27 µmol/L)	89 [27–178.3]
Urine oxalate-to-creatinine ratio (random) (mg/g) (ref.: <48 mg/g)	52 [35.0–189.0]
eGFR (mL/min/1.73 m^2^) at the time of diagnosis	70 [17–100]
eGFR (mL/min/1.73 m^2^) at the time of complications	22.5 [11–47]
**Current status**	
Alive	18 (85.7)
Deceased	3 (14.3)

PH: primary hyperoxaluria, *AGXT*: alanine–glyoxylate aminotransferase, *GRHPR*: glyoxylate and hydroxypyruvate reductase, UTI: urinary tract infection, CKD: chronic kidney disease, eGFR: estimated glomerular filtration rate, FTT: failure to thrive

The most frequently identified *AGXT* variant was NM_000030.3:c.33dup (p.Lys12GlnfsTer156), a pathogenic frameshift variant located in exon 1. This variant was detected in eight patients (38.1%), including six with Homozygous (Hom) genotypes and two with Heterozygous (Het) genotypes (Table 2).

**Table 2:** Genetic variants identified in 21 patients with primary hyperoxaluria

Gene	MANE transcript: nucleotide change	Amino acid change	Exon	ACMG class	Mutation type	Zygosity	Phenotypes	Management	Mortality	*n* (%)
*AGXT*	NM_000030.3:c.24_25insC	p.(Thr9HisfsTer159)	1	LP	Frameshift	Ho	Kidney stones, UTI, stage III CKD, hematuria	Conservative	No	1 (4.8)
	NM_000030.3:c.33dup	p.(Lys12GlnfsTer156)	1	P	Frameshift	5 Ho, 2 He	Kidney stones, UTI, stages II, III, and V CKD stage, nephrocalcinosis, FTT, hematuria	PD, HD, CRRT, LT, KT, conservative	No	8 (38.1)
	NM_000030.3:c.346G > A	p.(Gly116Arg)	2	P	Missense	Ho	Stage V CKD, nephrocalcinosis, FTT	PD, HD, CRRT, LT	Yes	1 (4.8)
	NM_000030.3:c.364 C > T	p.(Arg122Ter)	3	P	Nonsense	He	Absent	Conservative	No	1 (4.8)
	NM_000030.3):c.481G > A	p.(Gly161Ser)	4	P	Missense	Ho	Stage V CKD, FTT	PD, HD, CRRT, LT	Yes	1 (4.8)
	NM_000030.3:c.584T > G	p.(Met195Arg)	5	P	Missense	Ho	Kidney stones, stage V CKD, nephrocalcinosis, FTT, hematuria, hydronephrosis	PD, HD, CRRT, LT, conservative	No	4 (19.0)
	NM_000030.3:c.568G > C	p.(Gly190Arg)	5	P	Missense	Ho	Kidney stones, stage III CKD, nephrocalcinosis, hematuria, dysuria	Conservative	No	2 (9.5)
	NM_000030.3:c.680 + 3G > C	NA	Intron 6	VUS	Noncoding	Ho	Kidney stones, CKD stage II, nephrocalcinosis, FTT	LT	No	1 (4.8)
	Not documented	-	-	-	-	Ho	Kidney stones, UTI, CKD stage V, FTT, hematuria	PD, HD, CRRT, KT + LT	No	1 (4.8)
*GRHPR*	NM_012203.2:c.64G > A	p.(Ala22Thr)	1	VUS	Missense	He	Kidney stones	Conservative	No	1 (4.8)

MANE: Matched Annotation from NCBI and EMBL-EBI, ACMG: American College of Medical Genetics and Genomics classification, P: pathogenic, LP: likely pathogenic, VUS: variant of uncertain significance, He: heterozygous, Ho: homozygous, NA: not applicable, CKD: chronic kidney disease, UTI: urinary tract infection, FTT: failure to thrive, KT: kidney transplantation, LT: liver transplantation, PD: peritoneal dialysis, HD: hemodialysis

Regarding laboratory findings, the median serum oxalate level was markedly elevated at 89 µmol/L, substantially exceeding the normal reference range of 11–27 µmol/L. Similarly, the urinary oxalate-to-creatinine ratio was elevated (median, 52 mg/g; reference: <48 mg/g). The median estimated glomerular filtration rate (eGFR) at diagnosis was 70 mL/min/1.73 m^2^, which declined sharply to 22.5 mL/min/1.73 m^2^ at the time of complication, reflecting progressive renal impairment (Table 1).

Regarding clinical features, kidney stones represented the most common presenting feature (71.4%). Chronic kidney disease (CKD) was also prevalent, with 9.5%, 19%, and 42.9% of patients presenting with stages II, III, and V CKD, respectively. Other prevalent clinical features included nephrocalcinosis (47.6%), failure to thrive (38.1%), hematuria (33.3%), and urinary tract infections (14.3%). Notably, no patients had proteinuria, and dysuria was reported in only one patient (4.8%, Table 1).

**Fig. 2:**
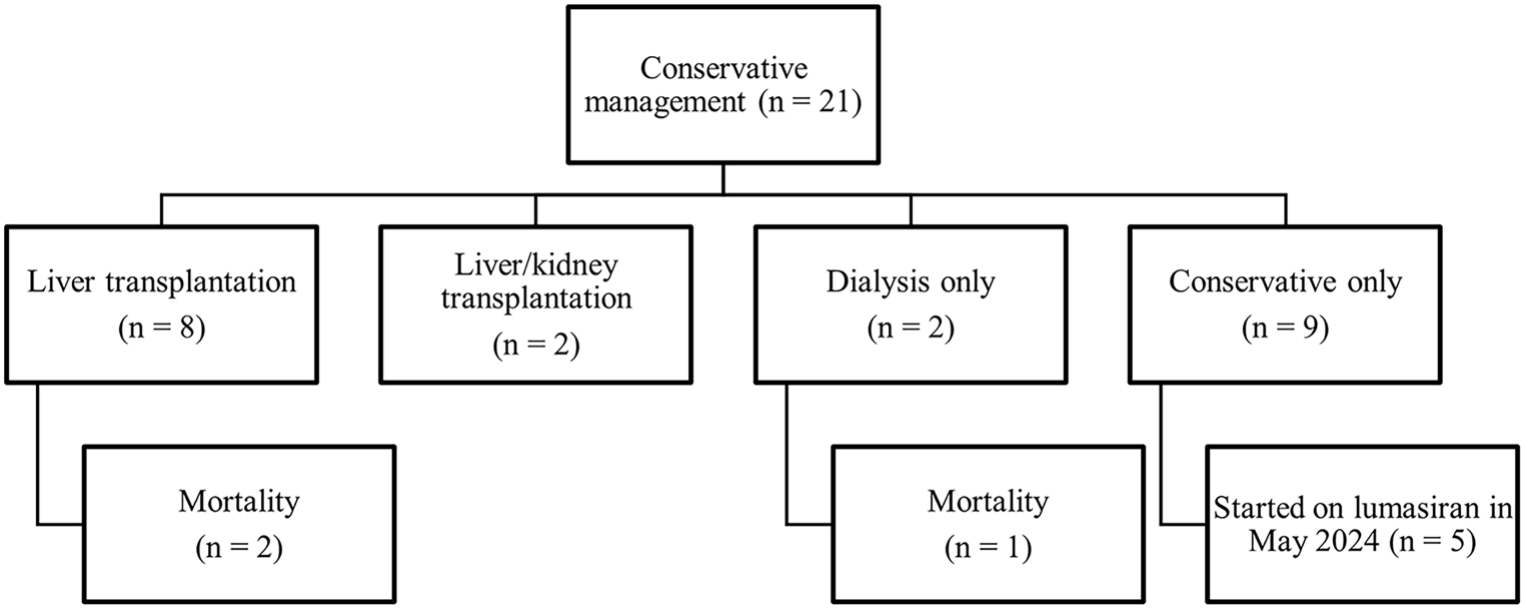
Treatment pathways and outcomes in pediatric patients with primary hyperoxaluria (2014–2023)

**Fig. 3:**
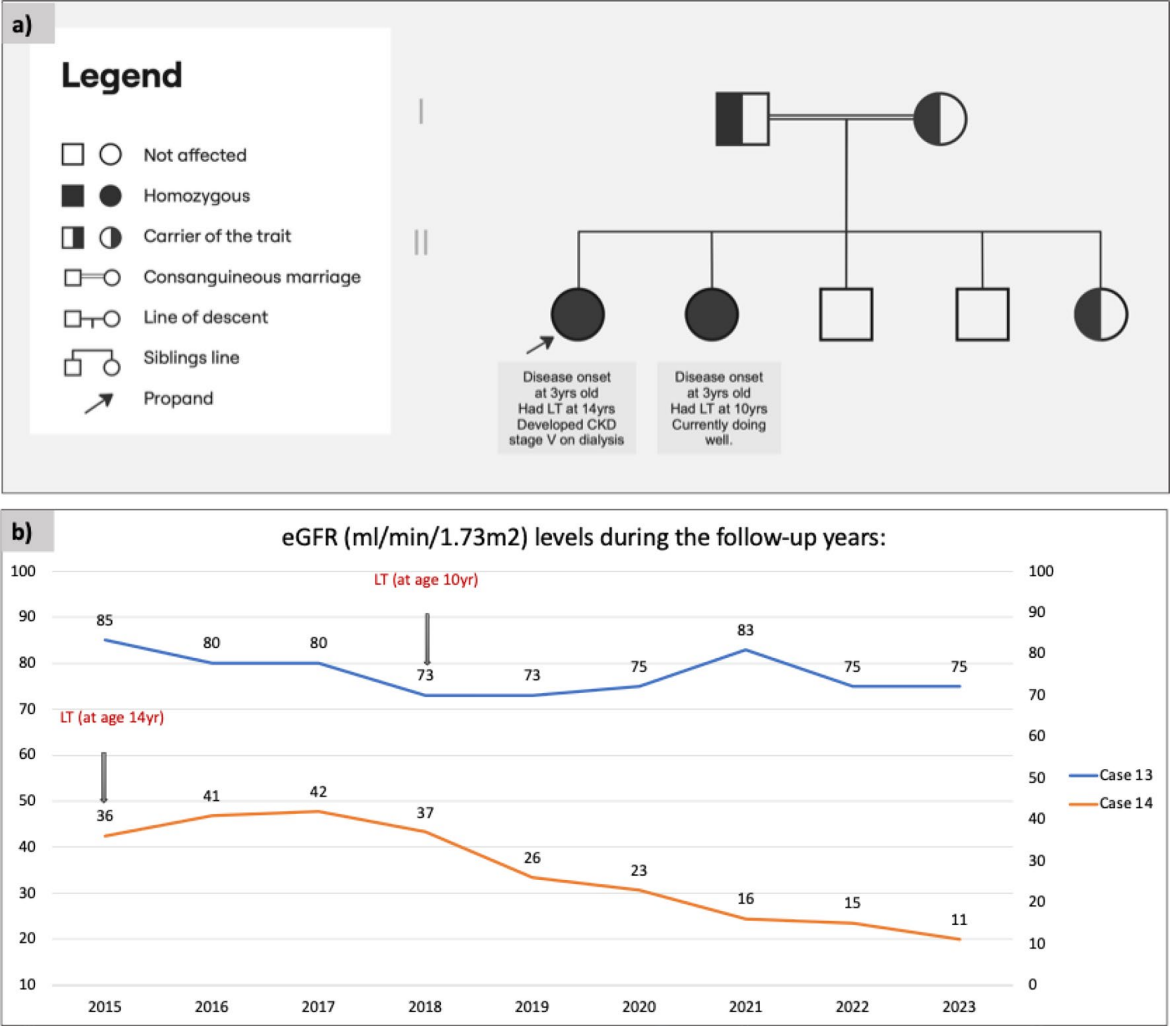
(**a**) Pedigree of a consanguineous family affected by primary hyperoxaluria type 1 because of an autosomal recessive alanine–glyoxylate aminotransferase gene mutation (c.33dup: p. Lys12GlnfsTer156). Both parents were heterozygous carriers. Among their five children, two females were affected (homozygous), one female was a carrier (heterozygous), and two males were unaffected noncarriers. (**b**) Liver transplantation (LT) outcomes and longitudinal estimated glomerular filtration rate (eGFR) trends in the two affected siblings (Cases 13 and 14). Case 13 underwent early LT at 10 years old, resulting in sustained improvement in renal function, as reflected by eGFR levels. She is currently 15 years old with stable kidney function. Case 14 underwent late LT at 14 years old. Despite the intervention, she progressed to stage V chronic kidney disease, and she is currently on dialysis at 22 years old

All patients received conservative management. In addition, 38.1% of patients also underwent liver transplantation, whereas combined liver–kidney transplantation was performed in 9.5% of patients (Fig. 2). Figure 3 highlights outcomes in two female siblings (Cases 13 and 14) with the same *AGXT* variant. Case 13, who received an early liver transplant at 10 years old, exhibited sustained clinical and renal improvement, avoiding progression to systemic oxalosis. Conversely, her sister (Case 14) underwent late liver transplantation at 14 years old. However, she progressed to stage V CKD, and she currently requires dialysis at age of 22 years old.

The overall mortality rate was 14.3%, with two of the three deceased patients having undergone liver transplantation (Table 1).

## Discussion

This case series presented the clinical, genetic, and management profiles of 21 pediatric patients diagnosed with PH across Saudi Arabia. Notably, most patients had PH1, which is predominantly linked to *AGXT* variants. This aligns with national data [12, 13] and international findings [17, 18] illustrating that PH1 is the most prevalent form of hereditary hyperoxaluria.

Consanguinity and positive family history were prominent features in this study, reflective of patterns commonly observed in autosomal recessive disorders within regions with high rates of consanguineous marriages, such as the Middle East [11]. These familial links both reinforce the genetic basis of PH and support the potential benefit of targeted genetic screening and counseling programs. For example, Hoppe et al. conducted a newborn screening study in Germany involving more than 77,000 infants and successfully identified potential carriers of PH1 and PH3 and detected symptomatic cases through follow-up [19]. These data support the feasibility and potential benefits of genomic newborn screening even in low-prevalence populations [19].

The most frequent variant in our series was *AGXT* variant NM_000030.3:c.33dup (p.Lys12GlnfsTer156). No patient with this variant died, supporting prior findings suggesting variable expressivity despite its pathogenicity [2, 12]. This variant, which has also been reported in Chinese populations [8], might represent a founder effect in Saudi Arabia, warranting inclusion in local diagnostic panels.

Other variants in *AGXT*, including NM_000030.3:c.481G >A (p.Gly161Ser) and NM_000030.3:c.346G >A (p.Gly116Arg), appeared to be associated with worse renal outcomes based on descriptive observations [20]; however, formal statistical testing was not conducted due to the small sample size. The NM_000030.3):c.481G >A(p.(Gly161Ser) variant is classified as pathogenic in multiple ClinVar submissions [20], and clinical data from the European Hyperoxaluria Consortium further support its potential association with poor renal prognosis in PH1 patients [21]. Similarly, the NM_000030.3: c.346G >A(p.(Gly116Arg) variant has been reported as pathogenic and associated with PH1 in several ClinVar submissions [22]. However, prognostication based on genotype remains limited because of the multifactorial nature of PH progression [21].

Interestingly, for family 4, one sibling (Case 6) carried a different Hom variant [NM_000030.3: c.24_25insC: p.(Thr9HisfsTer159)] than her sisters (Cases 7 and 8), who both shared the same Hom variant [NM_000030.3:c.33dup (p.Lys12GlnfsTer156)]. Because the parental genotypes were unknown, we could not determine the exact mechanism. However, it is likely that both parents carry these two mutations, probably making them compound Het.

Moreover, missense variants accounted for 42.9% of all identified variants, comparable to the findings of Alfadhel et al., who reported a rate of 48% in their Saudi cohort [12]. The novel *GRHPR* variant NM_012203.2:c.64G >A (p.Ala22Thr) was identified in one patient with PH2, contributing to the expanding variant spectrum and highlighting the need for functional validation studies [8].

Nephrolithiasis and nephrocalcinosis were the most prevalent clinical features, consistent with findings in other studies [8, 23–25]. Approximately 42.9% of patients progressed to CKD stage V, with median eGFR declining from 70 to 22.5 mL/min/1.73 m^2^, emphasizing the progressive nephrotoxicity of oxalate accumulation [2]. Notably, none of our patients had proteinuria or stage I or IV CKD, and developmental delay was not reported, contradicting previous findings [25, 26], possibly because our cohort received earlier diagnoses and improved supportive care.

The mortality rate in our series was 14.3%, in line with findings from India, where mortality or CKD was observed in 30% of patients, and around 20% of patients required surgical interventions, illustrating the severity of complications in advanced disease [27].

In terms of management, eight patients underwent liver transplantation, and two underwent combined liver–kidney transplantation. These procedures are well established in correcting the metabolic defect in PH1 [24, 28]. One patient in our study exhibited marked clinical improvement after early liver transplantation, preventing progression to systemic oxalosis (Fig. 3).

Importantly, lumasiran, a novel RNA interference (RNAi) therapy approved for PH1 [29], was initiated in six patients in May 2024. By targeting hepatic glycolate oxidase, lumasiran significantly reduces oxalate production, preserving renal function in early-stage patients. Early data from Saffe et al. revealed promising reductions in urinary oxalate levels after 6 months of therapy [30], although long-term outcomes remain under investigation. We are currently conducting a prospective study at our center to further assess its efficacy.

Conservative therapies, including hyperhydration and pyridoxine, were also utilized. Despite not being curative, these measures are essential for symptom management and delaying progression, particularly when implemented early [31]. These findings reinforce the importance of newborn screening and prompt family testing to facilitate timely intervention.

The most recent clinical practice guidelines by Groothoff et al. recommend genetic testing for PH1–3 as early as possible—ideally within 30 days—when a patient presents with suspected PH and significant kidney impairment (eGFR < 30 mL/min/1.73 m^2^). For patients with better renal function (eGFR >30 mL/min/1.73 m^2^), genetic confirmation should still be pursued promptly, but test processing times can vary by country. Additionally, genetic counseling is strongly recommended for couples who are both carriers of PH1-related mutations to support early detection and treatment planning for any affected children [32].

## Limitations

This study offers valuable insights into PH in Saudi children. However, it was limited by its small sample size and retrospective design.

## Conclusions

PH1 is the predominant form of hyperoxaluria among Saudi patients. The most frequent variant in the *AGXT* gene was NM_000030.3: c.33dup (p.Lys12GlnfsTer156), supporting its inclusion in local genetic panels. We observed a potential association between worse renal prognosis and other *AGXT* variants, specifically NM_000030.3: c.481G > Ap.(Gly161Ser) and NM_000030.3: c.346G > A p.(Gly116Arg), displayed potential relationships with worse renal prognosis.

Clinical complications such as nephrocalcinosis and CKD were common, necessitating close monitoring. Liver transplantation is a key therapeutic option in advanced cases, whereas novel RNAi-based therapies such as lumasiran offer promising outcomes for early intervention, potentially reducing the need for organ transplantation in patients with PH1. Overall, in this series, early diagnosis and intervention played a critical role in improving patient outcomes, as a notable survival rate was achieved despite the disease severity of the cohort.

## Supplementary Information

Below is the link to the electronic supplementary material.

Supplementary Material 1

Supplementary Material 2

## Data Availability

Data are available upon request.
