# Supplementary material for: Primary hyperoxaluria: insights into its clinical presentation, genetic mutations, and transplantation outcomes in a pediatric population in a tertiary care center
Source: Orphanet J Rare Dis. 2025 Oct 28;20:546. doi: 10.1186/s13023-025-04082-8 (PMC12570568; doi:10.1186/s13023-025-04082-8)
Supplement: Supplementary file 1 — Supplementary Material 1 [file 13023_2025_4082_MOESM1_ESM.docx]

**S1. Figure: a)** PH1 mechanism: Deficiency of AGT (AGXT gene) leads to glyoxylate accumulation and excess oxalate production. **b)** PH2 mechanism: GRHPR deficiency impairs glyoxylate and hydroxypyruvate metabolism, increasing oxalate and glycolate levels.
