# Supplementary material for: Primary hyperoxaluria: insights into its clinical presentation, genetic mutations, and transplantation outcomes in a pediatric population in a tertiary care center
Source: Orphanet J Rare Dis. 2025 Oct 28;20:546. doi: 10.1186/s13023-025-04082-8 (PMC12570568; doi:10.1186/s13023-025-04082-8)
Supplement: Supplementary file 2 — Supplementary Material 2 [file 13023_2025_4082_MOESM2_ESM.docx]

**S2. Table:** Summary of individual clinical and genetic characteristics of patients with primary hyperoxaluria (n = 21).

| ID | Fam-ID | Gender (current age, years) | Age at diagnosis (months) | FH | Consanguinity | Mutation | Zygosity | Diagnosis on admission | eGFR at diagnosis | eGFR at the time of complications | Treatment | Outcome |
| --- | --- | --- | --- | --- | --- | --- | --- | --- | --- | --- | --- | --- |
| 1 | 1 | F (5) | 19 | + | - | *AGXT*: c.346G>A: p.(Gly116Arg) | Ho | Stage V CKD, nephrocalcinosis, FTT | 17 | 12 | PD/HD/CRRT, LT | Deceased |
| 2 | 2 | F (8) | 36 | + | + | *AGXT*: c.584T>G: p.(Met195Arg) | Ho | Kidney stones | 99 | 100 | Conservative | Alive |
| 3 | 2 | M (4) | 6 | + | + | *AGXT*: c.584T>G: p.(Met195Arg) | Ho | Kidney stones, hydronephrosis, hematuria | 100 | 100 | Conservative | Alive |
| 4 | 3 | M (16) | 120 | + | + | *AGXT*: c.568G>C: p.(Gly190Arg) | Ho | Kidney stone, stage III CKD, hematuria | 100 | 50 | Conservative | Alive |
| 5 | 3 | F (11) | 132 | + | + | *AGXT*: c.568G>C: p.(Gly190Arg) | Ho | Kidney stones, nephrocalcinosis, hematuria, dysuria | 88 | 34 | Conservative | Alive |
| 6 | 4 | F (11) | 60 | + | + | *AGXT*: c.24_25insC: p.(Thr9HisfsTer159) | Ho | Kidney stones, UTI, stage III CKD, hematuria | 100 | 40 | Conservative | Alive |
| 7 | 4 | F (6) | 6 | + | + | *AGXT*: c.33dup:  p.(Lys12GlnfsTer156) | Ho | Stage V CKD, nephrocalcinosis, FTT | 8 | 8 | PD/HD/CRRT | Died |
| 8 | 4 | F (22) | 168 | + | + | *AGXT*: c.33dup:  p.(Lys12GlnfsTer156) | Ho | Kidney stones, stage V CKD, nephrocalcinosis | 50 | 44 | PD/HD/CRRT, LT | Alive |
| 9 | 5 | M (13) | 6 | + | + | *AGXT*: c.584T>G: p.(Met195Arg) | Ho | Kidney stone, hydronephrosis | 100 | 66 | Conservative | M (13) |
| 10 | 5 | F (12) | 2 | + | + | *AGXT*: c.584T>G: p.(Met195Arg) | Ho | Kidney stones, stage V CKD, nephrocalcinosis, FTT, hematuria | 4 | 12 | PD/HD/CRRT, LT | Alive |
| 11 | 6 | F (10) | 36 | + | + | *AGXT*: c.33dup:  p.(Lys12GlnfsTer156) | Ho | Kidney stones, stage III CKD, nephrocalcinosis | 100 | 30 | PD/HD/CRRT, LT | Alive |
| 12 | 6 | M (9) | 96 | + | + | *AGXT*: c.33dup:  p.(Lys12GlnfsTer156) | Ho | Kidney stones, stage III CKD | 70 | 60 | Conservative | Alive |
| 13 | 7 | F (15) | 36 | + | + | *AGXT*: c.33dup:  p.(Lys12GlnfsTer156) | Ho | Kidney stones, stage II CKD, nephrocalcinosis, FTT, hematuria | 88 | 59 | LT | Alive |
| 14 | 7 | F (22) | 132 | + | + | *AGXT*: c.33dup:  p.(Lys12GlnfsTer156) | He | UTI, stage V CKD, nephrocalcinosis | 27 | 11 | LT | Alive |
| 15 | 8 | M (6) | 6 | − | + | *AGXT*: c.680+3G>C | Ho | Kidney stones, stage II CKD, nephrocalcinosis, FTT | 68 | 93 | LT | Alive |
| 16 | 9 | M (13) | 0 | + | + | *AGXT*: c.364C>T(p. Arg122*) | He | Absent | 100 | 100 | Conservative | Alive |
| 17 | 10 | M (8) | 36 | − | − | *GRHPR*: c.64G>A (p. Ala22Thr) | He | Kidney stones | 100 | 100 | Conservative | Alive |
| 18 | 11 | M (12) | 84 | − | + | *AGXT:* variant unknown | Ho | Kidney stones, UTI, stage V CKD, FTT, hematuria | 8 | 8 | PD/HD/CRRT, KT, LT | Alive |
| 19 | 12 | M (5) | 3 | − | + | *AGXT*: c.481G>A: p.(Gly161Ser) | Ho | Stage V CKD, FTT | 5 | 5 | PD/HD/CRRT, LT | Deceased |
| 20 | 13 | M (8) | 12 | + | + | *AGXT*: c.33dup:  p.(Lys12GlnfsTer156) | Ho | Stage V CKD | 14 | 15 | KT, LT | Alive |
| 21 | 14 | F (6) | 36 | + | + | *AGXT*: c.33dup:  p.(Lys12GlnfsTer156) | He | Kidney stones, stage V CKD, nephrocalcinosis, FTT | 50 | 11 | PD/HD/CRRT | Alive |

*AGXT*: alanine–glyoxylate aminotransferase, CRRT: continuous renal replacement therapy, CKD: chronic kidney disease, eGFR: estimated glomerular filtration rate, FH: family history, FTT: failure to thrive, *GRHPR*: glyoxylate and hydroxypyruvate reductase, HD: hemodialysis, He: heterozygous, Ho: homozygous, KT: kidney transplantation, LT: liver transplantation, PD: peritoneal dialysis, UTI: urinary tract infection.
